# 3-Hydr­oxy-3-nitro­methyl­indolin-2-one

**DOI:** 10.1107/S1600536809039087

**Published:** 2009-10-03

**Authors:** Ying Tang, Gang Chen, Jie Zhang, Shijun Chen

**Affiliations:** a Xi’an Shiyou University, College of Chemistry and Chemical Engineering, Dianzi’er Road No. 18 Xi’an 710065, Xi’an, People’s Republic of China

## Abstract

In the title compound, C_9_H_8_N_2_O_4_, the indolin-2-one ring system is substanti­ally planar [maximum deviation = 0.0353 (15) Å]. In the crystal structure, inter­molecular N—H⋯O and O—H⋯O hydrogen bonds are responsible for the formation of a three-dimensional network.

## Related literature

For the synthesis of the title compound, see: Imre *et al.* (2001 ▶); Long *et al.* (1978 ▶).
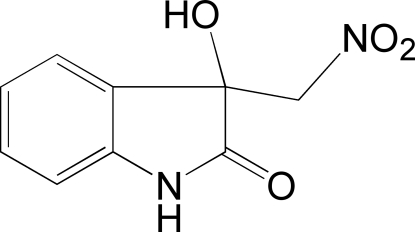


## Experimental

### 

#### Crystal data

C_9_H_8_N_2_O_4_
*M* *_r_* = 208.17
Orthorhombic, 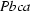

*a* = 10.515 (2) Å
*b* = 7.3736 (14) Å
*c* = 23.261 (4) Å
*V* = 1803.6 (6) Å^3^
*Z* = 8
Mo *K*α radiation
μ = 0.12 mm^−1^
*T* = 293 K
0.21 × 0.18 × 0.15 mm

#### Data collection

Bruker SMART CCD area-detector diffractometer
Absorption correction: multi-scan (*SADABS*; Bruker, 2002 ▶) *T*_min_ = 0.941, *T*_max_ = 0.961
16322 measured reflections
3807 independent reflections
2098 reflections with *I* > 2σ(*I*)
*R*_int_ = 0.026

#### Refinement

*R*[*F* ^2^ > 2σ(*F* ^2^)] = 0.045
*wR*(*F* ^2^) = 0.135
*S* = 1.03
3807 reflections
136 parameters
H-atom parameters constrained
Δρ_max_ = 0.31 e Å^−3^
Δρ_min_ = −0.21 e Å^−3^

### 

Data collection: *SMART* (Bruker, 2002 ▶); cell refinement: *SAINT* (Bruker, 2002 ▶); data reduction: *SAINT*; program(s) used to solve structure: *SHELXS97* (Sheldrick, 2008 ▶); program(s) used to refine structure: *SHELXL97* (Sheldrick, 2008 ▶); molecular graphics: *ORTEP-3* (Farrugia, 1997 ▶) and *DIAMOND* Brandenburg (1999 ▶); software used to prepare material for publication: *WinGX* (Farrugia, 1999 ▶).

## Supplementary Material

Crystal structure: contains datablocks I, global. DOI: 10.1107/S1600536809039087/rz2364sup1.cif

Structure factors: contains datablocks I. DOI: 10.1107/S1600536809039087/rz2364Isup2.hkl

Additional supplementary materials:  crystallographic information; 3D view; checkCIF report

## Figures and Tables

**Table 1:** Hydrogen-bond geometry (Å, °)

*D*—H⋯*A*	*D*—H	H⋯*A*	*D*⋯*A*	*D*—H⋯*A*
N1—H1*A*⋯O2^i^	0.86	2.13	2.9849 (14)	171
O2—H2*A*⋯O1^ii^	0.82	1.93	2.7408 (13)	171

Symmetry codes: (i) 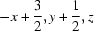; (ii) 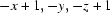.

**Fig. 1.**
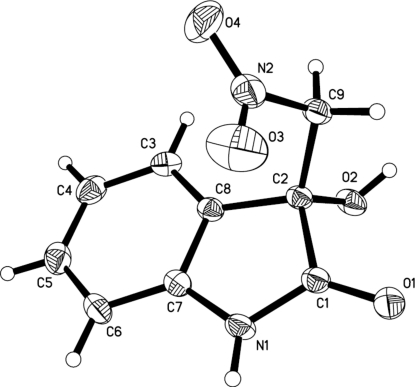
An ORTEP-3 drawing of the title compound, with the atom-numbering scheme and 30% probability displacement ellipsoids.

**Fig. 2.**
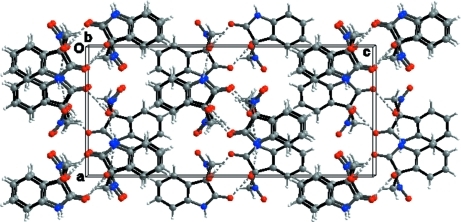
Packing diagram of the title compound viewed approximately along the b axis. Dashed lines indicate hydrogen bonds.

## supplementary crystallographic information

### Comment

3-Hydroxy-3-nitromethyl-1,3-dihydro-indolin-2-one, an important intermediate for the synthesis of natural products, has been synthesized by Henry reaction (Imre *et al.*,2001; Long *et al.*, 1978). Dehydration of this compound as well as its derivatives provides 3-nitromethylene-1,3-dihydro-indolin-2-one, which is used as a dipolarophile in 1,3-dipolar cycloadditon reactions to synthesize spiro-oxindole compounds. In this paper we report the X-ray crystal structure of the title compound.

The X-ray structural analysis confirmed the assignment of the structure of the title compound from spectroscopic data. The molecular structure is depicted in Fig. 1, and a packing diagram of is depicted in Fig. 2. Geometric parameters of the title compound are in the usual ranges. The indolin-2-one ring system is substantially planar, with a maximum deviation of 0.0353 (15) Å for atom C4. In the crystal structure, intermolecular N–H···O and O–H···O hydrogen bonds (Table 1) are effective in the stabilization of the structure and are responsible for the formation of a three-dimensional network. The O atoms of nitro group are not involved in any hydrogen bond.

### Experimental

Isatin (1 mmol) was dissolved in nitromethane (20 ml), catalyzed by DBU, until the disappearance of the starting material, as evidenced by thin-layer chromatography. The solvent was removed *in vacuo* and the residue was separated by column chromatography (silica gel, petroleum ether/ethyl acetate = 5:1 v/v), giving the title compound. ^1^H-NMR (D_6_—DMSO, 400 MHz): 10.56 (1*H*, s), 7.39 (1*H*, d, J = 7.2 Hz), 7.26 (1*H*, td, J = 7.6, 1.2 Hz), 6.98 (1*H*, t, J = 7.6 Hz), 6.85 (1*H*, d, J = 7.6 Hz), 6.75 (1*H*, s), 4.99 (2*H*, dd, J = 12.8, 8.0 Hz); ^13^C-NMR (CDCl_3_, 100 MHz): 176.0, 142.6, 130.3, 127.9, 124.7, 121.9, 110.1, 78.5, 72.8; MS (EI) m/*z*: 208 (*M*^+^). 30 mg of the solid compound was dissolved in methanol (30 ml) and the solution was kept at room temperature for 4 d. Slow evaporation of the solvent gave colourless single crystals suitable for X-ray analysis.

### Refinement

All H atoms were positioned geometrically, with C–H = 0.93–0.97 Å, O–H = 0.82 Å, N–H = 0.86 Å, and refined using riding model, with *U*_iso_(H) = 1.2*U*_eq_(C, N) or 1.5*U*_eq_(O).

### Crystal data

**Table:**

C_9_H_8_N_2_O_4_	*F*(000) = 864
*M**_r_* = 208.17	*D*_x_ = 1.533 Mg m^−^^3^
Orthorhombic, *P**b**c**a*	Mo *K*α radiation, λ = 0.71073 Å
Hall symbol: -P 2ac 2ab	Cell parameters from 3122 reflections
*a* = 10.515 (2) Å	θ = 1.8–34.3°
*b* = 7.3736 (14) Å	µ = 0.12 mm^−^^1^
*c* = 23.261 (4) Å	*T* = 293 K
*V* = 1803.6 (6) Å^3^	Block, colourless
*Z* = 8	0.21 × 0.18 × 0.15 mm

### Data collection

**Table:**

Bruker SMART CCD area-detector diffractometer	3807 independent reflections
Radiation source: fine-focus sealed tube	2098 reflections with *I* > 2σ(*I*)
graphite	*R*_int_ = 0.026
φ and ω scans	θ_max_ = 34.3°, θ_min_ = 1.8°
Absorption correction: multi-scan (*SADABS*; Bruker, 2002)	*h* = −12→12
*T*_min_ = 0.941, *T*_max_ = 0.961	*k* = −9→11
16322 measured reflections	*l* = −28→36

### Refinement

**Table:**

Refinement on *F*^2^	Primary atom site location: structure-invariant direct methods
Least-squares matrix: full	Secondary atom site location: difference Fourier map
*R*[*F*^2^ > 2σ(*F*^2^)] = 0.045	Hydrogen site location: inferred from neighbouring sites
*wR*(*F*^2^) = 0.135	H-atom parameters constrained
*S* = 1.03	*w* = 1/[σ^2^(*F*_o_^2^) + (0.0699*P*)^2^ + 0.2564*P*] where *P* = (*F*_o_^2^ + 2*F*_c_^2^)/3
3807 reflections	(Δ/σ)_max_ = 0.001
136 parameters	Δρ_max_ = 0.31 e Å^−^^3^
0 restraints	Δρ_min_ = −0.21 e Å^−^^3^

### Special details

**Table:**

Geometry. All e.s.d.'s (except the e.s.d. in the dihedral angle between two l.s. planes) are estimated using the full covariance matrix. The cell e.s.d.'s are taken into account individually in the estimation of e.s.d.'s in distances, angles and torsion angles; correlations between e.s.d.'s in cell parameters are only used when they are defined by crystal symmetry. An approximate (isotropic) treatment of cell e.s.d.'s is used for estimating e.s.d.'s involving l.s. planes.
Refinement. Refinement of *F*^2^ against ALL reflections. The weighted *R*-factor *wR* and goodness of fit *S* are based on *F*^2^, conventional *R*-factors *R* are based on *F*, with *F* set to zero for negative *F*^2^. The threshold expression of *F*^2^ > σ(*F*^2^) is used only for calculating *R*-factors(gt) *etc*. and is not relevant to the choice of reflections for refinement. *R*-factors based on *F*^2^ are statistically about twice as large as those based on *F*, and *R*- factors based on ALL data will be even larger.

### Fractional atomic coordinates and isotropic or equivalent isotropic displacement parameters (Å^2^)

**Table:**

	*x*	*y*	*z*	*U*_iso_*/*U*_eq_	
N1	0.73793 (10)	0.22536 (14)	0.59575 (4)	0.0345 (2)	
H1A	0.8121	0.2684	0.5882	0.041*	
C7	0.68870 (11)	0.20320 (15)	0.65183 (5)	0.0313 (3)	
C8	0.56744 (11)	0.12898 (14)	0.64887 (5)	0.0294 (2)	
C2	0.53211 (11)	0.10262 (14)	0.58677 (5)	0.0287 (2)	
O1	0.67140 (9)	0.16574 (14)	0.50346 (4)	0.0452 (3)	
C3	0.50087 (12)	0.08802 (17)	0.69844 (5)	0.0374 (3)	
H3A	0.4196	0.0386	0.6966	0.045*	
C9	0.41221 (12)	0.20580 (16)	0.56828 (5)	0.0354 (3)	
H9A	0.4059	0.2048	0.5267	0.043*	
H9B	0.3377	0.1453	0.5837	0.043*	
C6	0.74609 (13)	0.24268 (17)	0.70380 (5)	0.0396 (3)	
H6A	0.8264	0.2951	0.7055	0.048*	
N2	0.41524 (12)	0.39586 (15)	0.58894 (5)	0.0470 (3)	
C5	0.67832 (15)	0.20049 (18)	0.75336 (6)	0.0450 (3)	
H5A	0.7145	0.2253	0.7890	0.054*	
C4	0.55872 (14)	0.1227 (2)	0.75123 (5)	0.0448 (3)	
H4A	0.5167	0.0934	0.7852	0.054*	
O3	0.50508 (14)	0.48843 (17)	0.57450 (9)	0.0904 (5)	
O4	0.32881 (14)	0.44827 (19)	0.61941 (6)	0.0776 (4)	
C1	0.65394 (11)	0.17044 (15)	0.55545 (5)	0.0317 (3)	
O2	0.51621 (8)	−0.08414 (11)	0.57414 (4)	0.0391 (2)	
H2A	0.4586	−0.0966	0.5507	0.059*	

### Atomic displacement parameters (Å^2^)

**Table:**

	*U*^11^	*U*^22^	*U*^33^	*U*^12^	*U*^13^	*U*^23^
N1	0.0244 (5)	0.0384 (5)	0.0408 (5)	−0.0081 (4)	−0.0017 (4)	0.0000 (4)
C7	0.0299 (6)	0.0267 (5)	0.0372 (6)	0.0003 (4)	−0.0040 (4)	0.0000 (4)
C8	0.0259 (6)	0.0268 (5)	0.0353 (5)	0.0019 (4)	−0.0030 (4)	0.0005 (4)
C2	0.0237 (6)	0.0254 (5)	0.0370 (5)	−0.0008 (4)	−0.0022 (4)	−0.0024 (4)
O1	0.0378 (5)	0.0603 (6)	0.0374 (5)	−0.0092 (4)	0.0023 (4)	−0.0040 (4)
C3	0.0303 (6)	0.0389 (6)	0.0431 (6)	0.0043 (5)	0.0031 (5)	0.0048 (5)
C9	0.0276 (6)	0.0352 (6)	0.0435 (6)	0.0037 (5)	−0.0069 (5)	−0.0055 (5)
C6	0.0370 (7)	0.0356 (6)	0.0463 (6)	−0.0006 (5)	−0.0139 (5)	−0.0037 (5)
N2	0.0474 (7)	0.0366 (6)	0.0569 (7)	0.0135 (5)	−0.0168 (5)	−0.0048 (5)
C5	0.0520 (8)	0.0454 (7)	0.0377 (6)	0.0136 (6)	−0.0120 (5)	−0.0052 (5)
C4	0.0476 (8)	0.0503 (7)	0.0364 (6)	0.0154 (6)	0.0037 (5)	0.0044 (5)
O3	0.0736 (9)	0.0354 (6)	0.1620 (17)	−0.0061 (6)	−0.0100 (9)	0.0024 (7)
O4	0.0866 (10)	0.0763 (8)	0.0700 (8)	0.0389 (7)	−0.0037 (7)	−0.0255 (7)
C1	0.0262 (6)	0.0314 (5)	0.0374 (6)	−0.0017 (4)	−0.0008 (4)	−0.0019 (4)
O2	0.0317 (5)	0.0265 (4)	0.0591 (5)	−0.0010 (3)	−0.0098 (4)	−0.0076 (3)

### Geometric parameters (Å, °)

**Table:**

N1—C1	1.3500 (16)	C3—H3A	0.9300
N1—C7	1.4130 (16)	C9—N2	1.4819 (17)
N1—H1A	0.8600	C9—H9A	0.9700
C7—C6	1.3822 (17)	C9—H9B	0.9700
C7—C8	1.3893 (17)	C6—C5	1.390 (2)
C8—C3	1.3823 (17)	C6—H6A	0.9300
C8—C2	1.5042 (15)	N2—O3	1.2129 (19)
C2—O2	1.4180 (13)	N2—O4	1.2155 (18)
C2—C9	1.5340 (16)	C5—C4	1.383 (2)
C2—C1	1.5563 (16)	C5—H5A	0.9300
O1—C1	1.2237 (14)	C4—H4A	0.9300
C3—C4	1.3940 (19)	O2—H2A	0.8200
			
C1—N1—C7	111.51 (10)	C2—C9—H9A	109.4
C1—N1—H1A	124.2	N2—C9—H9B	109.4
C7—N1—H1A	124.2	C2—C9—H9B	109.4
C6—C7—C8	121.81 (11)	H9A—C9—H9B	108.0
C6—C7—N1	128.55 (11)	C7—C6—C5	117.02 (12)
C8—C7—N1	109.63 (10)	C7—C6—H6A	121.5
C3—C8—C7	120.63 (11)	C5—C6—H6A	121.5
C3—C8—C2	130.37 (11)	O3—N2—O4	124.39 (14)
C7—C8—C2	108.98 (10)	O3—N2—C9	117.33 (13)
O2—C2—C8	110.71 (9)	O4—N2—C9	118.28 (14)
O2—C2—C9	109.07 (9)	C4—C5—C6	121.94 (12)
C8—C2—C9	114.09 (9)	C4—C5—H5A	119.0
O2—C2—C1	108.19 (9)	C6—C5—H5A	119.0
C8—C2—C1	101.81 (9)	C5—C4—C3	120.29 (12)
C9—C2—C1	112.70 (9)	C5—C4—H4A	119.9
C8—C3—C4	118.28 (12)	C3—C4—H4A	119.9
C8—C3—H3A	120.9	O1—C1—N1	126.62 (11)
C4—C3—H3A	120.9	O1—C1—C2	125.24 (10)
N2—C9—C2	111.13 (9)	N1—C1—C2	108.05 (10)
N2—C9—H9A	109.4	C2—O2—H2A	109.5

### Hydrogen-bond geometry (Å, °)

**Table:**

*D*—H···*A*	*D*—H	H···*A*	*D*···*A*	*D*—H···*A*
N1—H1A···O2^i^	0.86	2.13	2.9849 (14)	171
O2—H2A···O1^ii^	0.82	1.93	2.7408 (13)	171

Symmetry codes: (i) −*x*+3/2, *y*+1/2, *z*; (ii) −*x*+1, −*y*, −*z*+1.
